# The Significance of Newly Derived Disease Activity Parameters Neutrophil/Albumin Ratio, Neutrophil/Complement C3 Ratio, and Albumin/Globulin Ratio in a Group of Patients With Lupus Nephritis

**DOI:** 10.7759/cureus.94377

**Published:** 2025-10-12

**Authors:** Violeta Rabrenovic, Milorad Rabrenovic, Milica Petrovic, Dejan Pilcevic, Boban Labovic, Nemanja Rancic

**Affiliations:** 1 Clinic of Nephrology, Military Medical Academy, Belgrade, SRB; 2 University of Defense, Medical Faculty of the Military Medical Academy, Belgrade, SRB; 3 Center for Hyperbaric Medicine, Military Medical Academy, Belgrade, SRB; 4 Clinic of Neurology, Military Medical Academy, Belgrade, SRB; 5 Center for Clinical Pharmacology, University of Defense, Medical Faculty of the Military Medical Academy, Belgrade, SRB

**Keywords:** activity, complement c3, globulin, lupus nephritis, neutrophil

## Abstract

Introduction: Lupus nephritis (LN) is the most serious manifestation of systemic lupus erythematosus (SLE), which worsens the course and prognosis of this autoimmune disease. The standard assays we use to assess LN activity still do not have sufficient sensitivity. The aim of this study was to determine the parameters neutrophil/albumin ratio (NAR), neutrophil/complement C3 ratio (NC3r), and albumin/globulin ratio (AGR) in patients with LN, as well as the significance of these parameters in comparison with the standard parameters that we use to determine the activity of lupus nephritis.

Methods: Of the total number of subjects (72 patients) with lupus nephritis (mean age: 43.62 ± 14.38 years), who were included in this study, 50% (36) of the patients had active disease (group A-LN), and the other half had disease in remission (group R-LN). In addition to standard laboratory parameters, we also determined the derived parameters NAR, NC3r, and AGR.

Results: In the group comparison, a significant difference was observed in the following parameters: albumin, complement C3, antinuclear antibody (ANA), anti-double-stranded DNA antibody (anti-ds-DNA Ab), proteinuria g/24h (proteins from 24 hours collected urine), and Systemic Lupus Erythematosus Activity Index/renal (SLEDAI/r) (p < 0.000). Correlation of NAR with total proteins (r = -0.359, p = 0.003), albumin (r = -0.590, p < 0.001), SLEDAI/r (r = 0.460, p < 0.001), and proteinuria g/24h (r = 0.515, p < 0.001) was significant in the A-LN group. Correlation of NC3r with total proteins (r = -0.340, p = 0.003), albumin (r = -0.584, p < 0.001), C3 (r = -0.474, p < 0.001), anti-ds-DNA Ab (r = 0.283, p = 0.019), proteinuria g/24h (r = 0.586, p < 0.001), and SLEDAI/r (r = 0.559, p < 0.001) was significant in the A-LN group. AGR in the A-LN group significantly correlated with albumin (r = 0.581, p < 0.001), C3 (r = 0.376, p = 0.023), and SLEDAI/r score (r = -0.354, p = 0.036). According to the coordinates on the receiver operating characteristic (ROC) curve for NAR, the cutoff value is 0.104, the sensitivity is 72.2%, and the specificity is 66.7%; for parameter NC3r, the cutoff value is 4.849, the sensitivity is 83.3%, and the specificity is 69.4%; and for AGR, the cutoff value is -1.608, the sensitivity is 30.6%, and the specificity is 63.9%.

Conclusion: Our data show a significant relationship between NAR, NC3r, and AGR and lupus activity. NC3r has proven to be the most significant in providing valuable data for identifying patients with active LN and as a simple indicator in clinical practice.

## Introduction

Despite the fact that the diagnosis of lupus nephritis (LN), as the most serious manifestation of systemic lupus erythematosus (SLE), has been significantly facilitated, the problem of monitoring disease activity, potential relapses, and the progression of kidney lesions is still relevant [1]. Lupus nephritis (LN) represents one of the most severe manifestations of SLE, and markers of early activity as well as highly sensitive indicators of exacerbation, and thus opportunities for timely changes in therapy are still insufficient. These conditions not only affect the severity and progression of the renal lesion but also sometimes affect the patient's survival.

Many studies have been published examining different biomarkers and entire palettes of biomarkers, but still no answer has been found in the form of a simple and practically applicable indicator of LN activity, bearing in mind that the standard parameters are insufficient [2-5]. This was precisely the reason for determining the derived parameters, which are a combination of analysis available in practice, and which could be significant in the evaluation of LN activities. Neutrophil/albumin ratio (NAR), neutrophil/complement C3 ratio (NC3r), and albumin/globulin ratio (AGR) are combined immune-inflammatory-nutritional markers that have been studied in many diseases and can be significant in determining the activity of LN [6-13].

NAR, as a marker representing the ratio of neutrophils to albumin, indicates systemic inflammation and malnutrition and, as such, is determined in many malignant and autoimmune diseases. Bearing in mind its simplicity and availability in everyday practice, studies were published that indicated the importance of determining NAR as a predictor of cardiovascular comorbidity in patients on peritoneal dialysis, in diabetes, and in the progression of chronic kidney failure, but there are very few studies in which the NAR was determined in patients with lupus nephritis [14-16].

In contrast to NAR, there are a couple of studies describing the NC3r marker, which, in addition to neutrophils as indicators of the immune response, also includes the level of complement C3 as an indicator of LN activity [9,10]. The significant sensitivity and specificity of this derived parameter are described, as well as the association with kidney involvement and retinal vasculopathy in SLE [9,10].

AGR indicates the ratio of albumin to globulin, and lower values ​​usually indicate a worse prognosis because hypoalbuminemia in LN correlates with active disease and a higher pathohistological class [11-13].

The objective of this study was to determine the parameters NAR, NC3r, and AGR, and their relationship with standard parameters of LN activity in our group of patients with LN.

## Materials and methods

The study included 72 patients with LN (13 (18.05%) men and 59 (81.94%) women, mean age: 43.62 ± 14.38 years) above 18 years of age, and with established SLE and LN diagnosis. Patients were diagnosed with SLE and LN according to the criteria of the American College of Rheumatology (ACR) and the revised World Health Organization (WHO) criteria by the International Society of Nephrology and the Renal Pathology Society (ISN/RPS) [17-19]. Within the Systemic Lupus Erythematosus Activity Index (SLEDAI 2000), the Systemic Lupus Erythematosus Activity Index/renal (SLEDAI/r) was used, which includes four criteria for assessing kidney damage [19].

The patients were divided into two groups: group LN active form (A-LN), with 36 (50%) patients, and group LN in remission (R-LN), with 36 (50%) patients. Group A-LN included patients with proteinuria ≥ 0.5 g/24h, SLEDAI/r criteria > 4, hypocomplementemia (C3 and C4), positive antibodies against double-stranded DNA (anti-ds-DNA Ab), and pathohistological findings of kidney biopsy. The group in remission is defined according to the following criteria: proteinuria ≤ 0.5 g/24h, SLEDAI/r criteria < 4, negative anti-ds-DNA antibodies, complement C3 and C4 within the reference range, and glomerular filtration rate (GFR) ≥ 60 mL/min/1.73 m^2^. All patients had glomerular filtration rate (GFR) ≥ 60 mL/min/1.73 m^2^, according to the Chronic Kidney Disease Epidemiology Collaboration (CKD-EPI) [20].

The study excluded patients with infectious syndrome, renal insufficiency (chronic kidney disease (CKD) and GFR < 60 mL/min/1.73 m^2^), malignant diseases, other autoimmune diseases, hematological diseases, and repeated transfusions, or patients who had previously received corticosteroids for other reasons.

Among the other characteristics of the research, we mention the following. Blood specimens were collected after an overnight fast. To avoid the influence of immunosuppressive therapy on laboratory parameters, in group A-LN, laboratory parameters were determined before the start of immunosuppressive therapy. Group R-LN received maintenance therapy: 5-10 mg/day of corticosteroids and 50-75 mg/day of azathioprine.

We determined the following laboratory parameters in all patients: C-reactive protein (CRP), complete blood count, creatinine, GFR, total proteins, and albumin. The following immunological tests were determined: complement C3 and C4, antinuclear antibodies (ANA), and antibodies against double-stranded deoxyribonucleic acid (anti-dsDNA Ab). From the urinary analyses, the following were monitored: urine sediment, SLEDAI/r, proteinuria g/24h (proteins from 24 hours of collected urine), and urine culture.

Morning fasting samples (at 6:00 a.m.) were analyzed before taking regular therapy. The devices for determining blood parameters were the ADVIA 120 Hematology System (Siemens Healthineers, Forchheim, Germany) and the Automatic Hematology Analyzer MACCURA F800 (Maccura Biotechnology Co., Ltd., Chengdu, China). The devices that were used to determine biochemical parameters were Auto-chemistry Analyzer DIRUI CS-2000 ADVIA 1800 (DIRUI Industrial Co., Ltd., Jilin, China) and the Dimension RxL Max Integrated Chemistry System (Siemens Healthineers).

We also determined the following parameters: NAR, NC3r, and AGR. Neutrophil/albumin ratio (NAR) was defined as neutrophil (109/L) divided by albumin (g/L). The ratio of neutrophil/complement C3 (NC3r) was defined as neutrophil divided by C3. The albumin/globulin ratio (AGR) is calculated by the following formula: albumin / (total protein - albumin). AGR ≤ 1 is significant.

The study was conducted according to the provisions of the Declaration of Helsinki and approved by the Ethics Committee of our institution (oral informed consent was obtained from all patients).

Statistical analysis

Data were analyzed using the Statistical Package for the Social Sciences version 26.0 (IBM Corp., Armonk, NY). Categorical variables were presented as frequencies and were analyzed using the Chi-square test. All continuous variables are presented as median (interquartile range: 25th-75th percentile) or mean ± standard deviation for data that are not normally or normally distributed, respectively. The Kolmogorov-Smirnov test was used to test the normality of data distribution. For intergroup comparisons, the Mann-Whitney test for non-parametric variables and the independent samples t-test for parametric data distribution were used. Spearman's correlation coefficient tested the relationship between variables. Optimal thresholds (cutoffs) of index values (NAR, NC3r, and AGR) for assessment of LN activity were determined by receiver operating characteristic (ROC) curve analysis. Statistical significance was defined as p < 0.05 for all comparisons.

## Results

The study included a total of 72 patients with LN, who were divided into two groups: the group with active LN (A-LN, n = 36) and the group with LN in remission (R-LN, n = 36). The basic clinical and laboratory characteristics of the patients with LN are shown in Table 1. Statistical significance between groups was obtained for parameters albumin, protein, C3, C4, ANA, anti-ds-DNA Ab, proteinuria g/24h, and SLEDAI/r score.

**Table 1:** Comparison between groups regarding baseline clinical and laboratory data Data are presented as mean ± standard deviation or median with interquartile range, depending on the normality of the data distribution. *Independent samples t-test #Mann-Whitney test Significant value is p < 0.05. BMI: body mass index, CRP: C-reactive protein, GFR: glomerular filtration rate, C3: complement C3, C4: complement C4, ANA: antinuclear antibodies, anti-ds-DNA Ab: antibodies against double-stranded deoxyribonucleic acid, proteinuria g/24h: proteins from 24 hours collected urine, SLEDAI/r: Systemic Lupus Erythematosus Activity Index/renal

Parameters	A-LN (n = 36)	R-LN (n = 36)	t/U	p-value
Age (years)	41.05 ± 16.14	46.19 ± 12.06	1.530	0.065*
BMI (kg/m^2^)	23.92 ± 4.85	28.90 ± 4.55	0.055	0.480*
CRP (mg/L)	3.47 (3.01-5.70)	3.30 (3.00-4.07)	496.0	0.087#
Creatinine (umol/L)	80.50 (65.25-130.75)	82.50 (67.25-106.50)	609.0	0.660#
GFR (mL/min/1.73 m^2^)	77.64 ± 34.14	80.24 ± 27.39	0.356	0.723*
Albumin (g/L)	32.00 ± 7.09	40.81 ± 4.07	6.456	<0.001*
Proteins (g/L)	57.94 ± 9.26	68.33 ± 7.36	5.268	<0.001*
C3 (g/L)	0.64 ± 0.20	0.91 ± 0.16	6.270	<0.001*
C4 (g/L)	0.10 ± 0.06	0.15 ± 0.05	3.457	0.001*
ANA (IU/L)	3.00 (2.00-3.00)	0.00 (0.00-1.00)	170.0	<0.001#
Anti-dsDNA Ab (IU/mL)	90.00 (45.00-149.00)	15.00 (15.00-20.00)	157.5	<0.001#
Proteinuria (g/24h)	3.55 (1.79-4.66)	0.25 (0.16-0.37)	2.0	<0.001#
SLEDAI/r score	6.00 (3.25-7.00)	0.00 (0.00-1.00)	25.0	<0.001#

Age, gender distribution, and BMI did not show a significant difference between the groups, as did CRP and kidney function parameters, creatinine and GFR. Comparisons of the two groups according to the values ​​of the derived parameters NAR, NC3r, and AGR are shown in Table 2. A statistically significant difference was obtained for all three parameters, with NC3r having the highest significance (p ≤ 0.001). NAR and NC3r values ​​were higher in the group with active disease, while AGR was lower in the group with active LN compared to the group in remission.

**Table 2:** Comparison between groups regarding NAR, NC3r, and AGR Bold values are significant. Values are shown as mean ± standard deviation. *Independent samples t-test Significant value is p<0.05. NAR: neutrophil/albumin ratio, NC3r: neutrophil/complement C3 ratio, AGR: albumin/globulin ratio

Parameters	A-LN (n = 36)	R-LN (n = 36)	t	p-value
NAR	0.161 ± 0.093	0.105 ± 0.053	3.154	0.002
NC3r	8.581 ± 5.740	4.620 ± 2.061	3.897	<0.001
AGR	1.356 ± 0.529	1.556 ± 0.374	1.852	0.034

Correlations of standard parameters of lupus nephritis activity (creatinine, GFR, proteins, albumins, complement C3, C4, anti-ds-DNA antibodies, and SLEDAI/r score) with derived parameters NAR, NC3r, and AGR are shown in Table 3.

**Table 3:** Correlation of standard parameters of LN activity with derived parameters NAR, NC3r, and AGR Bold values are significant. Spearman's correlation coefficient Significant value is p < 0.05. CRP: C-reactive protein, GFR: glomerular filtration rate, C3: complement C3, C4: complement C4, ANA: antinuclear antibodies, anti-ds-DNA Ab: antibodies against double-stranded deoxyribonucleic acid, proteinuria g/24h: proteins from 24 hours collected urine, SLEDAI/r: Systemic Lupus Erythematosus Activity Index/renal, LN: lupus nephritis

	NAR		NC3r		AGR	
Spearman's rho	Correlation coefficient	Significance (two-tailed)	Correlation coefficient	Significance (two-tailed)	Correlation coefficient	Significance (two-tailed)
CRP (mg/dL)	0.110	0.358	0.054	0.652	0.194	0.256
Creatinine (umol/L)	0.208	0.079	0.223	0.06	0.022	0.895
GFR (mL/min/1.73 m^2^)	-0.122	0.306	-0.119	0.317	-0.068	0.694
Protein (g/L)	-0.359	0.002	-0.340	0.003	0.221	0.193
Albumin (g/L)	-0.590	<0.001	-0.584	<0.001	0.581	<0.001
C3 (g/L)	-0.169	0.156	-0.474	<0.001	0.376	0.023
C4 (g/L)	0.047	0.695	-0.156	0.191	0.170	0.319
Anti-ds-DNA Ab (IU/mL)	0.192	0.116	0.283	0.019	0.133	0.445
Proteinuria (g/24h)	0.515	<0.001	0.586	<0.001	-0.152	0.382
SLEDAI/r score	0.460	<0.001	0.559	<0.001	-0.354	0.036

**Figure 1:**
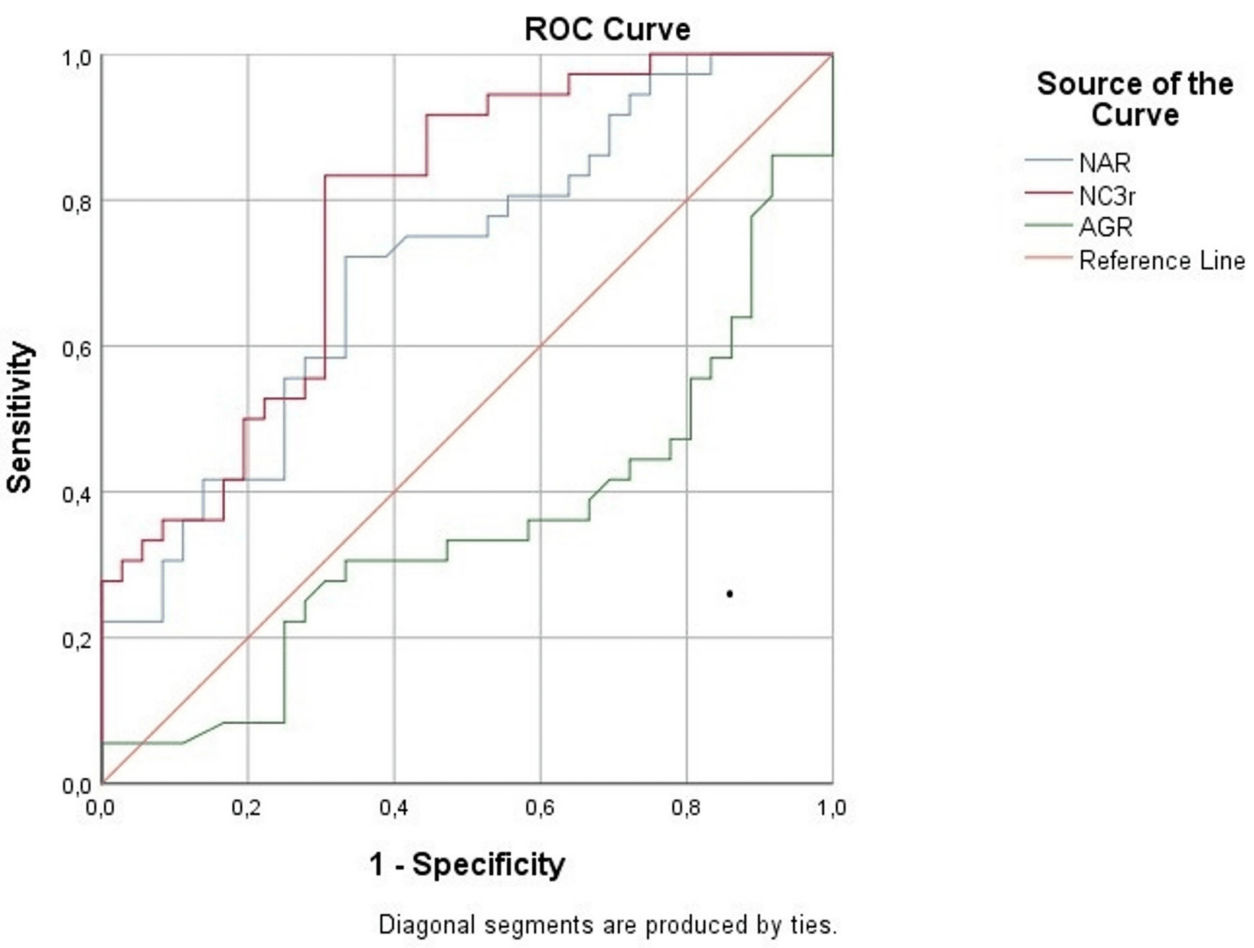
ROC curve analysis: ROC of NAR, NC3r, and AGR ROC: receiver operating characteristic, NAR: neutrophil/albumin ratio, NC3r: neutrophil/complement C3 ratio, AGR: albumin/globulin ratio

The correlation of NC3r with standard parameters of LN activity was significant for serum proteins (r = -0.340, p = 0.003), albumin (r = -0.584, p < 0.001), C3 (r = -0.474, p < 0.001), anti-ds-DNA Ab (r = 0.283, p = 0.019), proteinuria g/24h (r = 0.586, p < 0.001), and SLEDAI/r score (r = 0.559, p < 0.001). The correlation of AGR parameter was significant with albumin (r = 0.581, p < 0.001), C3 (r = 0.376, p = 0.023), and SLEDAI/r score (r = -0.354, p = 0.036). Comparing these three parameters, NC3r showed significant correlation with the most number of standard parameters. If the ROC analysis is performed to evaluate the clinical efficiency of the observed derived parameters, then it can be seen that all three parameters were shown to be significant with areas under the curve (AUC) greater than 0.7 for NAR and NC3r, while the area under the curve for AGR was clinically less significant (0.351). According to the coordinates on the ROC curve, the following results were obtained: for NAR, the cutoff value is 0.104, the sensitivity is 72.2%, and the specificity is 66.7%; for NC3r, the cutoff value is 4.849, the sensitivity is 83.3%, and the specificity is 69.4%; and for AGR, the cutoff value is 1.608, the sensitivity is 30.6%, and the specificity is 63.9%. The highest sensitivity with acceptable specificity was shown by NC3r based on the value of the area under the curve (AUC), and the lowest sensitivity on the ROC curve was shown by AGR (Table 4, Figure 1).

**Table 4:** Area under the ROC curve analysis of NAR, NC3r, and AGR Significant value is p < 0.05. ROC: receiver operating characteristic, NAR: neutrophil/albumin ratio, NC3r: neutrophil/complement C3 ratio, AGR: albumin/globulin ratio

Test result variable	Area	Asymptotic significance	Asymptotic 95% confidence interval		Cutoff value	Sensitivity	Specificity
			Lower bound	Upper bound			
NAR	0.710	0.002	0.592	0.828	0.104	72.200	66.700
NC3r	0.781	0.000	0.675	0.887	4.849	83.300	69.400
AGR	0.351	0.030	0.222	0.481	1.608	30.600	63.900

## Discussion

Lupus nephritis is manifested by very serious damage to all kidney structures (glomeruli, tubules, interstitium, and blood vessels), and it is believed that 10 years after the diagnosis, 10%-30% of patients will show end-stage kidney failure [21]. This is the reason for interest in facilitating and accelerating the diagnosis using easily available laboratory parameters. The significance of neutrophils, albumin, and complement C3 in the diagnosis, treatment, and follow-up of patients with lupus nephritis was the subject of studies conducted in these patients in previous years, and the formation of derived parameters that could be significant for the follow-up of these patients.

As an indicator of inflammation and poorer prognosis, NAR was described in studies that included patients with cancers, infections, and heart failure [7,8]. A smaller number of studies describe the determination of NAR in autoimmune diseases such as Behçet's disease (BD) and rheumatoid arthritis (RA) [6,22]. In a study by Egyptian authors, the research included patients with Behçet's disease (BD) and the connection between active disease and NAR [6]. Their results indicated a significant positive correlation of NAR with clinical and laboratory parameters of BD activity (active uveitis, arthralgia, and oral ulceration), as well as with parameters CRP, ESR, and CRP/albumin ratio (CAR) [6]. As a conclusion, the study stated that NAR, as an immuno-inflammatory parameter, can be useful in determining active BD. Similar results were published by Liu et al., who, in a study that included 38,272 subjects, compared the ratio of neutrophil percentage and albumin value (NPAR) in rheumatoid arthritis (RA) [22]. RA is a disease that is closely related to the pathogenesis and progression of the disease. An increase in NPAR as a ratio of increased neutrophil activity and a decrease in serum albumin also provided insight into RA activity [22].

Within other autoimmune diseases, there are not many studies that mention the relationship of NAR with SLE and LN. Kamal et al. investigated a group of patients with SLE and paid special attention to the association of NAR with bacterial infections in this group [23]. Their observation is that the increase in NAR is related to the infectious syndrome [23]. They concluded that NAR can indicate infection in the group of patients with SLE, and not SLE activity, because NAR correlated with parameters such as procalcitonin and neutrophil/lymphocyte ratio (NLR), while a very weak correlation was shown with the SLEDAI. Also, their study determined the sensitivity of NAR for infection in patients with SLE, which was 96.2%, and the specificity was 80.6% [23].

In our study, patients with LN were examined and divided into two groups: those with active disease and patients in remission. One of the criteria for inclusion in this study was that the patients did not have infectious complications. Our patients had a statistically significant difference between the groups in the NAR value, whereby the NAR in the group with active disease was higher (0.161 ± 0.093 versus 0.105 ± 0.053, p = 0.002). NAR correlated statistically significantly with standard parameters of disease activity: serum proteins, albumins, proteinuria g/24h, and SLEDAI/r score. Also, in our patients, a cutoff value of 0.104 was obtained, whereby the sensitivity was 72%, and the specificity was 66.7%. The results of our study are similar to the results of a Chinese study conducted by Li et al., who observed that NAR in the group with SLE and LN had statistically significantly higher values ​​compared to the group with SLE without LN (0.14 ± 0.01 versus 0.09 ± 0.01, p < 0.001) [24]. They did not observe significant differences for CRP, and due to the study design, patients with infection were not included in the study. A positive correlation with complement C3 was also established for the NAR parameter [24].

Neutrophils are also significant for the parameter that determines their relationship with complement C3. An increase in NC3r values ​​has been observed in active SLE, especially in LN; there are a couple of studies that indicate this [9,10]. The activity of complement C3 and its reduced level are typical of the activity of immune processes that are the essence of SLE and active nephritis. In our study, we found that NC3r was elevated in the group with active LN and that this difference was significant. A statistically significant correlation of NC3r was obtained for serum proteins, albumins, complement C3, anti-ds-DNA antibodies, urinary parameters for proteinuria g/24h, and SLEDAI/r score. By forming the ROC curve, a sensitivity of 83.3% and a specificity of 69.4% were obtained, while the cutoff value was 4.84.

Yu et al. published a study comparing NC3r in patients with SLE with active disease in both remission and healthy controls [9]. Their conclusion was the same as in our study: that NC3r was elevated in the group with active SLE, statistically significantly compared to the group of patients without active SLE and to the group of healthy subjects [9]. ROC curve analysis indicated that the cutoff value indicating disease activity was 5.93, with a sensitivity that was somewhat lower compared to our study (75.9%), as well as specificity (67%) [9]. Maitiyaer et al., in a study that included 220 patients with SLE who were divided into two groups according to whether they had LN or not, indicated a significant increase in NC3r values ​​in the group with LN [10]. The correlation between NC3r and proteinuria g/24h, and SLEDAI/r score was positively significant, and the optimal cutoff value was 6.40, with a sensitivity of 48.1% and a specificity of 72% [10].

Hypoalbuminemia always represents a very bad prognostic parameter, bearing in mind that it is observed in malignant diseases, inflammatory-immunological diseases, and other disorders of the nutritional status of patients [25-30]. It is precisely the disturbance of the ratio of albumin and globulin, expressed through the derived parameter AGR, that can represent a marker that indicates malnutrition, liver diseases, infections, and kidney diseases. The value of AGR is usually disturbed in states of dehydration, immunodeficiency, and genetic diseases.

In our group of patients with active LN, AGR was expectedly lower compared to the group in remission, and this difference was statistically significant. Also, AGR significantly correlated with parameters of active disease: complement C3 and SLEDAI/r. According to the ROC curve for AGR, the threshold value was 1.608, where the sensitivity was 30.6% and the specificity was 63.9%. In comparison with NAR and NC3r, the AGR parameter was still the least sensitive and specific according to the results of our study. In a retrospective study by Chinese authors, which included a total of 101 patients with SLE and 75 healthy individuals, AGR was examined and its correlation with parameters of active disease. They indicated that a reduced value of AGR is an inflammatory marker of active disease [11]. Serel et al., in a study that included a group of 109 patients with SLE and LN, tried to indicate the association of AGR for different classes of LN [12]. They observed a negative correlation between AGR and different classes of LN, where AGR showed a sensitivity of 95.8%, a specificity of 78.8%, and a cutoff value of 1.10 [12]. They conclude that AGR is a good predictor of LN progression [12]. Similar results were published by Liu et al. [13]. They published a study that included 194 patients with SLE, without renal involvement, who were followed for a period of 53.87 months, in which it was concluded that patients with low AGR, lower CRP, and elevated anti-ds-DNA antibodies with anamnestic data on alopecia develop LN more often, and these parameters represent significant predictors of SLE progression and LN development [13]. According to our study, all three derived parameters represent simple, accessible, useful, and easily applicable parameters in everyday practice that showed a good correlation with standard parameters of active disease.

Limitations

This study has several limitations that must be acknowledged. The limitations of this study were primarily related to the fact that these are the results of a single center, so more studies (multicenter) would be needed to confirm our results. Also, the number of subjects is a limitation, so future studies on a larger sample could indicate more affirmative results regarding the use of these markers as indicators of lupus nephritis activity.

## Conclusions

In conclusion, the results of this study suggest noninvasive, simple, and useful parameters that may indicate LN activity. Our data show a significant relationship between NAR, NC3r, and AGR, and lupus activity. NC3r has proven to be the most significant in providing valuable data for identifying patients with active LN and as a simple indicator in clinical practice.
